# Cognate reflex prediction as hypothesis test for a genealogical relation between the Panoan and Takanan language families

**DOI:** 10.1038/s41598-024-82515-3

**Published:** 2024-12-24

**Authors:** Frederic Blum, Carlos Barrientos, Adriano Ingunza, Johann-Mattis List

**Affiliations:** 1 Max-Planck Institute for Evolutionary Anthropology, Department of Linguistic and Cultural Evolution, 04301 Leipzig, Germany; 2 University of Passau, Chair for Multilingual Computerlinguistics, 94032 Passau, Germany; 3 Pontificia Universidad Catolica del Peru, Lima, 15088 Peru

**Keywords:** Human behaviour, Psychology

## Abstract

**Abstract:**

We present a novel approach for testing genealogical relations between language families. Our method, which has previously only been applied to closely related languages, makes predictions for cognate reflexes based on the regularity of proposed sound correspondences between language families that are hypothesized to be related. We test the hypothesis about a genealogical relation between Panoan and Takanan, two linguistic families of the Amazon. The workflow contributes to new ideas of hypothesis testing in historical linguistics and can likely be transferred to other language families. We predict 206 cognate reflexes from Shipibo-Konibo, a Panoan language, from independently proposed Proto-Takanan reconstructions and test our predictions in elicitation sessions with speakers of the language. We found 21 correct predictions from the core-set, as well as another 20 correct predictions from the extended set of predictions. In addition to confirming the previously established sound correspondence patterns, we find further evidence for additional patterns that suggest the reconstruction of three new phonemes for Proto-Pano-Takanan.

**Protocol registration:**

The stage 1 protocol for this Registered Report was accepted in principle on 06/05/24. The protocol, as accepted by the journal, can be found at: 10.17605/OSF.IO/FGBM7.

## Introduction

South America is one of the world’s areas with the largest linguistic diversity^1,2^. Despite many popular hypotheses for deep genealogical relations between language families on the continent^3–6^, few long-distance relationships are accepted among scholars, and much of the linguistic history of South America is unknown. We present a novel approach for testing genealogical relations in linguistics that makes predictions for cognate reflexes based on the regularity of proposed sound correspondences between language families that are hypothesized to be related, a method that has previously been applied to closely related languages^7,8^. The workflow contributes to new possibilities of hypothesis testing in historical linguistics and can likely be transferred to other language families. We test the hypothesis about a genealogical relation between Panoan and Takanan, two language families of the Amazon. Our predictions are based on sound correspondences from 44 lexical items that have been proposed by a previous study carried out independently^9^. If the assumption about the common descent of Panoan and Takanan is correct, we predict to find more evidence in favor of the earlier proposed sound correspondences in form of correctly predicted words.

In classical historical linguistics, genealogical relations between languages are analyzed using the ‘Comparative Method’^10–13^. This long-proven technique makes use of the regularity of sound change between related languages. In practice, this means that if language A and language B descend from the same ancestral proto-language, sound Y in language A will, in a specified phonological context from the reconstructed proto-language, always correspond to sound X in language B. The words which exhibit such sound correspondences are assumed to be ‘cognate’, having descended from the same ancestral word in an ancestral language. Cognate word forms can be used to ‘reconstruct’ ancestral word forms in the ancestral proto-language by explaining differences in the extant reflexes of individual cognate sets with the help of sound laws. Sound laws are rule-based instances of sound change in which an ancestral sound occurring in a certain number of words in the proto-language is turned into a new sound in the descendant language in all the cases where the ancestral sound occurs in a certain conditioning environment^14^. During this process of sound change, individual sounds in the word forms of the ancestral language are systematically modified, yielding the individual forms attested today.

For Proto-Panoan^15^ and Proto-Takanan^16^, more than 500 of such lexical reconstructions have been proposed. For Proto-Pano-Takanan, however, only 44 lexical and morphological items have been reconstructed^9^. A previous reconstruction of $\sim$120 words by Girard is not generally accepted, as the Panoan data used by the author is considered incomplete^15,17^. For this reason, the common descent of Panoan and Takanan is still not accepted as proven beyond reasonable doubt for some scholars and remains a hypothesis, albeit one with considerable evidence in favor. Our goal is to test this hypothesis with a new method that makes use of computational tools, while adhering to the comparative method. At the same time, we aim at contributing novel ideas to test genealogical relations in linguistics in general.

Cognate reflex prediction has so far primarily been used to predict words from closely related languages^7^. The general idea is to have a cognate set (the set of etymologically related words) and to predict the missing form in a language for which no reflex is attested in the cognate set. A variety of different methods has been tested for this purpose^8^. These include both expert-predictions, computer-assisted methods, and fully computational approaches. To the best of our current knowledge, only one study combined this method with predictions for fieldwork. Bodt & List showed how this method can be used to fill gaps from language documentation with efficient elicitation techniques^7^. Here, the cognate reflex predictions—which the authors shared in an official preregistration at an earlier stage^18,19^—were the starting point for eliciting words from speakers in different languages. In their study on Western Kho-Bwa, a branch of the Sino-Tibetan language family, they could elicit around 70% of their predictions, of which $\sim$70% were accurate in their phonemic shape. The other 30% slightly deviated phonetically from the predicted form. In general, they showed how cognate reflex prediction can work as an elegant method for preparing targeted fieldwork that combines the strength of computational predictions with traditional linguistic methods.

Based on 535 lexical forms that have been reconstructed for Proto-Takanan^16^, we make supervised predictions for the reflexes of those reconstructions in Shipibo-Konibo, a Panoan language spoken in the Peruvian Amazon. This means that we predict words from a reconstructed language family—Proto-Takanan—into a spoken language that belongs to another language family, by means of a hypothetical reconstruction proposed for a super family spanning both Panoan and Takanan languages. Depending on how well the prediction of individual reflexes across language families work, we hope to provide evidence on the relationship between Panoan and Takanan. We build those predictions upon the sound correspondences of the 44-item reconstruction of Valenzuela and Zariquiey (henceforth V&Z). An example for such a prediction is given in Table 1, which presents the correspondences of */b/ $\sim$ /β/ and */i/ $\sim$ /i/ between Proto-Takanan and Shipibo-Konibo twice. These and other similar examples lead to the prediction that if the term */b i ʃ i/ ‘to throw’ has a reflex in Shipibo-Konibo, it’s form would be /β i s i/.

Following the predictions, we made recordings with speakers of Shipibo-Konibo. In those elicitation sessions, we analyze whether the predicted word is attested in the language, or not. The detailed procedure will be presented in the Analysis Plan. If two language families are indeed related, we expect a substantial number of matches between the predicted form and the attested form in Shipibo-Konibo.

**Table 1:** Recurring correspondences between Proto-Takana and Proto-Pano lead to explicit predictions for reflexes of forms reconstructed for Proto-Takanan that have not been reconstructed for Proto-Panoan.

Language	Meaning	Form	Meaning	Form	Meaning	Form
Proto-Takana	COME	b e	SKIN	b i t i	THROW	b i ʃ i
Proto-Pano	COME	β ɨ	SKIN	β i ts i	?	?
Shipibo-Konibo	COME	β ɨ	SKIN	β i tʃ i	?	*β i s i*

The reflexes are predicted for Shipibo-Konibo, a Panoan language.

Research Question: Are the Panoan and Takanan linguistic families genealogically related?

Hypothesis 0: Panoan and Takanan are two separate linguistic families.
Hypothesis 1: Panoan and Takanan are genetically related linguistic families that descend from the same ancestral language. The sound correspondences between them are regular and recur multiple times across a large set of vocabulary.

Predictions to accept Hypothesis 1: We find sufficient cognate reflexes that are correctly predicted (with 20 and more items being deemed sufficient for our study). We accept a correctly predicted form as a plausible case of semantic change if we find at least two cases of evidence for a colexification in two different language families using the CLICS3 database^20^, following the approach proposed in Blevins & Sprout^21^. Following common practice in historical linguistics, finding less than 20 correct predictions does not necessarily mean that we should assume that both languages are genealogically unrelated, but rather that any possible genetic relation between them cannot be inferred anymore using the currently available methodologies^22^.

## Methods

### Ethics information

The research complies with all relevant ethical regulations. The necessary ethics approval for sound recordings with human participants has been granted by the ethics commission of the Max-Planck Society (Application number 2023_05). The participants received a compensation of 100 Peruvian soles per recording. We asked for explicit written and oral informed consent for recording and publishing the data, with an opt-in for doing so non-anonymously.

### Study design

**Figure 1:**
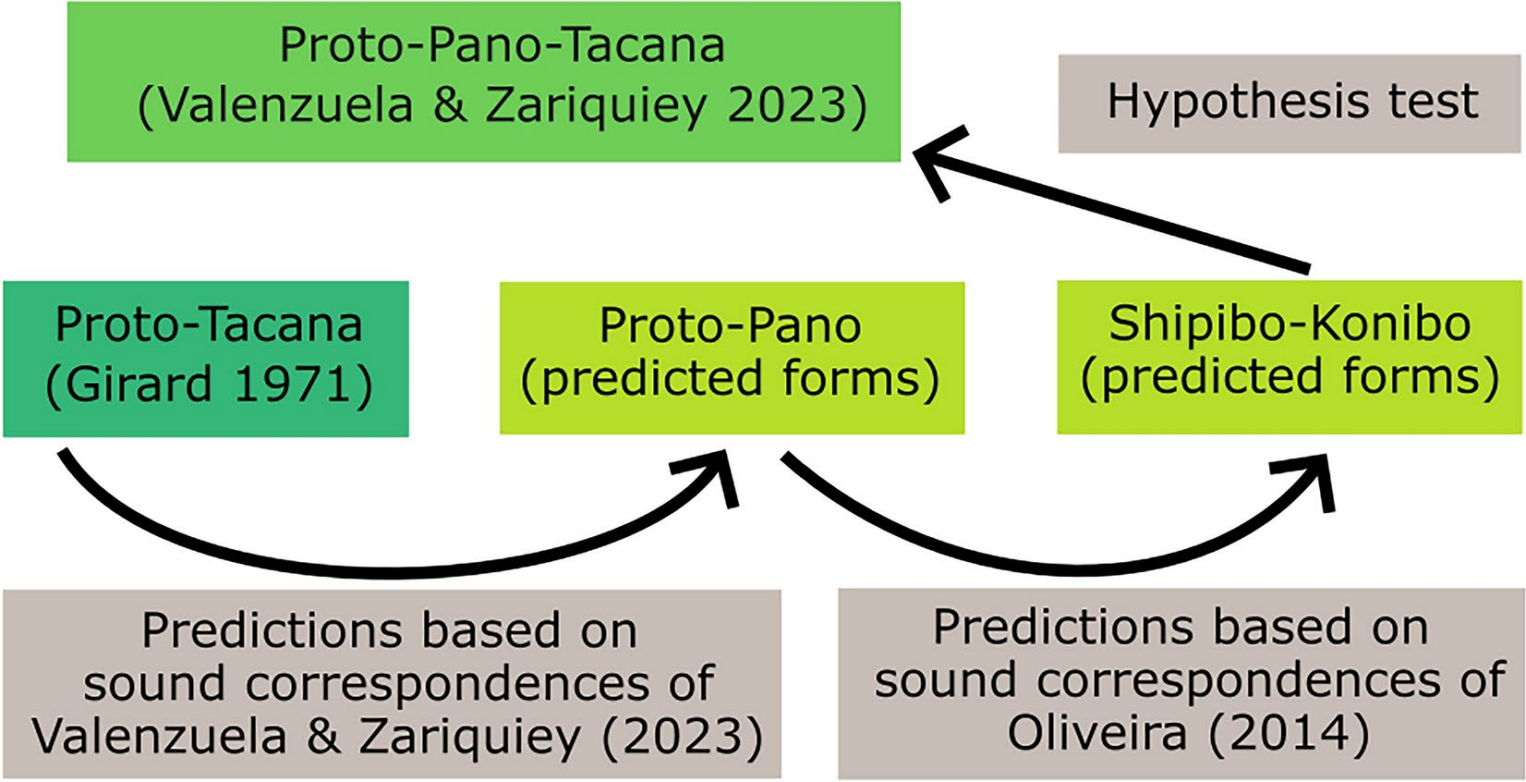
Workflow for predicting the cognate reflexes of Proto-Takanan forms in Shipibo-Konibo. The predictions are based on the reconstructions provided in the literature^9,15^. Grey boxes indicate the source for the sound correspondences used for making the predictions. The colored boxes in the center of the plot indicate the language families.

The general idea of our study was to predict cognate reflexes in Shipibo-Konibo, a Panoan language, for terms that have been reconstructed for Proto-Takanan. The starting point for our analysis are 535 lexical items of Proto-Takanan, the ancestral language to all Takanan languages, as reconstructed by Girard^16^. The first conversion step was based on the reconstruction of 44 lexical and morphological items for Proto-Pano-Takanan by Valenzuela and Zariquiey^9^, the hypothesized ancestor to both Panoan and Takanan languages^9^. Here, we converted Girard’s Proto-Takanan reconstructions to their stipulated form in Proto-Panoan, under the assumption that the sound correspondences between Proto-Panoan and Proto-Takanan have been correctly identified in the aforementioned study by V&Z. In the next step, the forms were converted to their reflexes in Shipibo-Konibo, a Panoan language spoken near Pucallpa, in the Peruvian Amazon. This step was based on the sound correspondences provided by Oliveira in his reconstruction of Proto-Pano^15^. We end up with the full list of predictions for Shipibo-Konibo, as well as their comparison to the original form in Proto-Takanan. The full workflow is visualized in Fig. 1.

The following information is directly relevant to our study:

- Original concept in Proto-Takanan (form X has meaning Y)
- Reconstruction for Proto-Takanan^16^
- Intermediate step: conversion into Proto-Panoan (our prediction)
- Prediction for Shipibo-Konibo (our prediction)
- Word for meaning Y (original concept) in Shipibo-Konibo (to be recorded)
- Information whether predicted form is attested in Shipibo-Konibo (to be recorded)
- Information on semantic or phonological change of the prediction to the actual attested word (to be analyzed)

The exact number of expected cognates is difficult to estimate, which has several reasons. For one, the rate of lexical change differs between language families^23^ and is difficult to estimate for a specific case. Second, even if the rate of lexical change could be reasonably well approximated, it is unclear at which time scale the split between Panoan and Takanan would be postulated. Even when looking at the Panoan family alone, the proposals for an initial divide between the languages ranges from 1500 years ago to 5000 years ago^17,24^. Similarly, no robust measures exist for estimating the probability of chance similarities between forms of two languages for the same word. Some computational approaches with this goal have been proposed in the form of permutation tests^25–27^, but they often contain statistical flaws that make their use inappropriate^22^. Even though computational permutation tests provide interesting new methods to test old hypotheses about language relationships^28,29^, the methods still lack a thorough verification through large-scale tests using generative models with simulated data. Furthermore, all those approaches run into the several problems related to Null Hypothesis Significance Testing and frequentist statistics in general^30,31^. Due to the lack of a general consensus for a viable method, we decided to go for a qualitative evaluation instead and look forward to new perspectives on computationally assessing the probability of chance similarities.

We implemented a simple string manipulation algorithm in Python to transform the Proto-Takanan reconstructions first to their stipulated forms in Proto-Pano, and from there to Shipibo-Konibo. The string replacements that have been used for making the predictions are provided in Table 7. If not otherwise specified, each sound correspondence is inferred directly from our manual alignments of the digitized version of the V&Z data. In some cases, there are one-to-many correspondences. Those cases are presented separated by a pipe ‘|’^32^, with the most frequent correspondence given first. In our elicitation sessions, we have asked for the combinations of the one-to-many correspondences, in case the first prediction does not hold up. Further details on this are given in the Analysis Plan. A special case are the fricatives */z/, */s/, and */j/, for which no clear correspondences could be identified. They are absent in V&Z’s study, but are proposed by Girard based on single cognates or expectations. Since we have observations of those fricatives in the Proto-Takanan data, we predict some reflexes for those segments based on those individual proposals, but have doubts as for the exact nature of those correspondences. Since it is estimated that it is necessary to have at least 300 or 400 items to find all the actual sound correspondences between two related languages^33,34^, the lack of examples for those segments is not too surprising, and we expect to find evidence for further sound correspondences that are not included in V&Z’s proposal during our analysis. These new sound correspondences would likely contribute to a more detailed reconstruction of Proto-Pano-Takanan.

**Table 2:** Correspondences between Proto-Takanan, Proto-Pano, and Shipibo.

Proto-Takana	Proto-Pano	Shipibo-Konibo	Comment
a	a	a	
⌃a	a | hi	a | hi	/hi/ only occurs in word-initial position
u	o	o	
o	o	o	
i	i | ɨ	i | ɨ	
e	ɨ	ɨ	
+	-	-	Morpheme separator
b	β	β	
d	r	n	
ɽ	r	n	Based on Girard^16^
r	n	n	word-finally sometimes dropped in Shipibo-Konibo
w	ɽ	ɽ	
k^w^	k^w^ | w	k | w	
k	k	k	
p	p	p	
m	m	m	
n	n	n	
ʈ ʂ	ʂ	ʂ	
t	t | ts	t | ts | tʃ	
ʃ	s	s	
z	ʃ	ʃ	Based on Girard, who gives only one example
s	s	s	Based on Girard, who gives no examples
j	s	s	Girard adds reflects /ʂ, ts/ without examples
ʔ	ʔ	ʔ	

Segments in brackets separated by pipes display one-to-many correspondences, with the most frequent reflex presented first.

By means of the pre-registration of the predictions evaluated in this study, we provided a new perspective on testing language relationships that combines computational and traditional methodology. The explicit prediction of cognate reflexes helps avoiding cherry-picking examples or fitting the data to our hypothesis. The result of the pilot study leads us to propose a minimum of 20 correct predictions. This threshold permits a robust evaluation of the hypothesis, and a discussion of the individual cases shows whether the sound correspondences can be upheld. We make the number of supporting cognate sets explicit for all sound correspondences.

### Pilot study

We test our prediction approach with two datasets: The Proto-Pano-Takanan reconstruction of V&Z^9^, and the Proto-Pano reconstruction of Oliveira^15^, which both include forms for Shipibo-Konibo. We digitized both datasets and converted them to CLDF^35,36^. Through linking the individual conceptlists used in each of the studies to Concepticon^37^, we can compute the intersection of concepts between those datasets and the Proto-Takanan dataset that we used for our predictions. As a result, we find that 15 of the 44 concepts in V&Z are also present in our predictions, and 119 concepts are present in both the Proto-Pano by Oliveira and the Proto-Takana reconstruction. Some of the concepts are present in both V&Z and Oliveira.

Since the data in V&Z has been used to propose the sound correspondences, we expect a large number of matches. Additionally, the dataset builds upon a core set of basic vocabulary that is designed to include a very low probability of lexical change, and already has a considerable degree of semantic change taken into account. An example for such a semantic change that is considered in the V&Z data is presented in Table 3. The colexification of SUN and MOON is confirmed in 38 languages of 18 language families in the CLICS3 database, fulfilling our criterion for the plausability of semantic change.

**Table 3:** Example for semantic change between Panoan and Takanan for the concepts SUN (Proto-Pano) and MOON (Proto-Takana).

Language	Concept	Form
Proto-Pano	SUN	β a r i
Proto-Takana	MOON	b a d i
Proto-Pano-Takana	MOON, SUN	b a d i

Both items descend from the same term **/b a d i/ in Proto-Pano-Takanan (V&Z).

As we show in Table 4, the expectations about a large amount of matches between attested form and predictions are fulfilled. For the V&Z dataset, we have an 86% of accuracy for the predictions, which is even higher than the accuracy reported in studies from closely related languages^7^, even though we have less matches (34%) . For the Proto-Panoan data, however, we have a considerably smaller intersection of concepts (21%) and accuracy ($\sim$16%). The attested matches partially overlap with each other across the datasets. Of the 17 matches for the reconstructed Proto-Pano dataset, 9 are also present in the more basic vocabulary of V&Z. The remaining matches (8 out of 95) gives us an estimate of the number of matches we can expect ($\sim$8–10%).

**Table 4:** Exact matches and matches with phonetic changes in each of the pilot datasets that have been cross-checked for our predictions.

Dataset	Intersection	Exact matches	Matches with phonetic changes	Total matches
V &Z	15/44 (34%)	7	6	13/15 (86%)
Oliveira	104/515 (21%)	7	10	17/104 (16%)

Considering the fact that we have explicit cognate judgements, there are some possible matches in our pilot study that include phonetic changes which deviate from our initially predicted forms. We present those in Table 5 to discuss whether they can be considered reflexes of the Proto-Takanan form or not. In the final analysis however, similar cases do not count towards our evaluation of the original hypothesis. We can summarize those cases into four different categories, all of which widespread in historical language change:

1. Loss of initial or final segments/syllables
2. One-to-many correspondences that are undetected so far
3. Sporadic sound changes that do not correspond regularly (e.g. /a/ to /ɨ/)
4. Sound correspondences with /ø/ in Proto-Takanan that are difficult to predict; they can be expected for elements like /h/ and /ʔ/, which can occur at various positions

**Table 5:** List of the phonetic changes attested in the pilot data, compared to the original prediction.

Dataset	Concept	Predicted	Attested	Type of irregular change
V&Z	TREE	[k | w] [i | ɨ]	h i w i	V&Z reconstruct this as */aki/ for Proto-Takanan (without providing reflexes), so this could be a case of /a/ to /hi/ a sound correspondence restricted to word-initial position, see Table 2
V&Z	BREAST	a ʂ o	ʂ o	Loss of initial vowel
V&Z	TONGUE	a n a	h a n a	Initial /h/ corresponds to /ø/ in Proto-Takana
V&Z	MOUTH	[k | w] a ʂ a	k ɨ ʂ a	Change of /a/ to /ɨ/
V&Z	TWO	β ɨ [t | ts | tʃ] a	r a β ɨ	Loss of syllables, only /β ɨ/ is shared
Oliveira	ARROW	p [i | ɨ] s a	p i a	Loss of intervocalic /s/?
Oliveira	BATHE	n a ɽ [i | ɨ]	n a ʃ i	Correspondence of /ʃ/ to Takanan is unclear this could either be an undetected correspondence, or an irregular change
Oliveira	MAIZE	s [i | ɨ] k ɨ	s ɨ k i	Possibly an undetected correspondence between /ɨ/ in Panoan and /i/ in Takanan
Oliveira	TIE	n [i | ɨ] s [i | ɨ]	n ɨ ʂ a	Unclear if indeed cognate
Oliveira	WIND	β ɨ n [i | ɨ]	n i w ɨ	Possible metathesis, confusion of /w/ and /β/

Analyzing the pilot data gives a first glance of possible irregular changes that we can expect for the main part of our study. It also helps us to consider the systematicity of apparent irregularities during the elicitation sessions. Furthermore, the verification of the sound correspondences based on published data shows that we can expect to make reasonable predictions for our experiment. In contrast to the pilot study, we only accepted such forms as correctly predicted that are formed out of fully regular sound correspondences. This includes the possibility of finding evidence for new regular sound correspondences (e.g. in specific phonological contexts) that have gone undetected in V&Z.

### Sampling plan

The wordlist by Girard is reduced to those entries that are unambiguously mapped in Concepticon^37^, which is the case for 324 of the 535 forms originally provided. The filtered entries are mostly grammatical morphemes, or terms for flora and fauna, which are often limited in their geographical distribution and prone to borrowing between languages. We also remove the concepts that have been used in the pilot study. Of the remaining 324 concepts, 15 are also part of the data used in the study of V&Z. Further, 119 of the 324 concepts are also reconstructed for Proto-Panoan^15^, of which 104 include the term as observerd in Shipibo-Konibo. Some of the concepts are present in both datasets. In total, 206 concepts remain that are not present in either study. Those concepts form the main list that were used for testing our hypothesis. When we had enough time during the recording sessions, we also elicited the data for the filtered concepts to search for matches with semantic changes which are not considered in the pilot analysis. But given the restricted semantic domains of the filtered concepts, we expect less matches than in our evaluation data. In case a speaker does not know the Shipibo-Konibo term for a certain concept, we consulted a different speaker of the language for that concept. The recordings will also be used to complement new resources on Pano-Takanan languages^38^.

### Analysis plan

The recordings have been made with speakers of the Shipibo-Konibo language. For each predicted form, we proceeded in three steps, as previously proposed by Bodt & List^7^:

1. Ask for the Shipibo-Konibo term for the source concept.
2. Ask if there are any other terms with a similar meaning.
3. Ask if there is a word equal to the form that was predicted.

Steps 1 and 2 aim at collecting the word that is used for each concept. As a reviewer pointed out, step 2 is highly dependent on the speaker, and what the speaker understands as ‘similar’ meaning. Our reasons to keep this step are two-fold: First, we want to include the (small) possibility of documenting possibly more archaic terms. Second, this allows us to stay close with the methodology of Bodt & List, for whom this step proved to be useful. Given the possibility of semantic change, we do not necessarily expect that the predicted form has the same meaning as its source in Proto-Takana. Due to this possibility, step 3 asks specifically for the predicted form, and records the meaning if such a word exists. If a plausible semantic relation between the source concept and this meaning can be established, we accept this as evidence in favor of the hypothesis. An example for semantic change has been presented earlier in Table 3. As made explicit when discussing the example, we accept a semantic change as plausible if colexifications for the two concepts in questions are attested in at least two separate language families.

Despite the role of regularity in historical linguistics, it has been acknowledged that there is no such thing as ‘complete regularity’ in sound correspondences. Many cases of sporadic sound change are attested. Those, however, are the exceptions to the so-called ‘sound laws’, and play only a minor role for reconstructing previous stages of a linguistic family. The exact amount of sporadic change and regularity is an open problem that has not yet been investigated quantitatively. For our study, this means that we do expect small amounts of phonological change in our predictions. However, in order to avoid any fitting of the data to our hypothesis, we did not accept those cases as correct predictions. Instead, we discuss in an exploratory analysis whether these cases can be used for further reconstruction of the proto-languages involved.

After the recordings, the data was transcribed manually using the software ELAN. The transcribed forms were then compared to the predicted form and inserted into the evaluation spreadsheet. For the evaluation, we consider three different cases. The predicted form can either be a) absent in Shipibo-Konibo (no evidence in favor of H1), b) be present, and lexify the same meaning as for Proto-Takanan, c) be present with the correct form, but a different meaning, for which a plausible case for semantic change can be made. In order to accept H1 as true, we expect at least 20 cases of true predictions (b and c).

## Results

In total, we have found 21 matches between attested forms and predictions from the core predictions in Shipibo-Konibo that present a clear semantic relation to the form in Proto-Takana. Albeit for one match, this surpasses our minimum threshold of 20 matches that we considered for confirming the genealogical relationship between Panoan and Takanan languages. To our surprise, we found way more direct semantic matches than expected. All of the matches either form a direct match, are direct verbalizations of a nominal concept (authority—‘to command’, hook—‘cast fish hook’), or have evidence for colexifications in the CLICS3 database^20^ (bitter and sour). The semantic fields from which the cognates come are widely spread and include body parts (e.g. nose, molar tooth), basic verb vocabulary (go, drink), and animals (e.g. toucan, porcupine). The full list of matches is presented in Table 6. The first part of the ID refers to the ID as presented in the paper, while the second number refers to the ID in the original list of predictions. Verbal morphemes /-ti/ and /-ati/ are separated by a dash. The forms that contain the new correspondence patterns are presented with a small superscript number in the table. The colexifications are presented with superscript letters.

**Table 6:** Correctly predicted forms with full phonetic regularity.

ID	Concept	Proto-Takana	Prediction	Form	Meaning
1-8	authority	w a ɽ a	ɽ a n a	ɽ a n a (- t i) ^1,2^	‘command’
2-15	bitter	p a ʈʂ e	p a ʂ ɨ	p a ɨ ^3^	‘sour’ ^A^
3-19	branch/twig	j a ʔ a	s a ʔ a	s a ʔ a (- t i)	‘divide in twigs’
4-27	carry	a b u	a β o	β o (- t i) ^4^	‘to carry in hand’
5-47	disease	n e r e	n ɨ n ɨ	n ɨ n ɨ	‘pain’, ‘to suffer’ ^B^
6-51	drink	i ʃ i	[i | ɨ] s [i | ɨ]	ʂ ɨ (- a t i) ^5,6^	‘drink’
7-72	fish poison	a ʈʂ a	[a | hi] ʂ a	a ʂ a	‘fish poison’
8-76	go	kʷ a	[k | w] a	k a (- t i)	‘to go’
9-81	grandfather	b a b a	β a β a	β a β a	‘grandson’ ^C^
10-92	hook	ʈʂ e w e	ʂ ɨ ɽ ɨ	ʂ ɨ r ɨ (- a t i)	‘cast fish hook’
11-102	light	u e	o ɨ	h o ɨ	‘light’
12-108	molar tooth	a m a k a	[a | hi] m a k a	m a k a (tʃ i p o) ^4^	‘molar (-tooth) ’
13-117	nose	w i	ɽ [i | ɨ]	ɽ ɨ (- k i n) ^1^	‘nose’
14-127	persuade	a m e r e	[a | hi] m ɨ n ɨ	m ɨ n i (- t i) ^4,7^	‘to give (advice)’
15-131	porcupine	i ʃ a	[i | ɨ] s a	i s a	‘porcupine’
16-132	pull	ɽ i ɽ e	n [i | ɨ] n ɨ	n i n i (- t i) ^2,7^	‘to pull’
17-163	sow seeds	b a n a	β a na	β a n a (- t i)	‘to sow seeds’
18-179	toucan	ʈʂ u kʷ e	ʂ o [k | w] ɨ	ʂ o k ɨ	‘toucan (species)’
19-180	toucan	p i ʈʂ a	p [i | ɨ] ʂ a	p i s a	‘toucan (species)’
20-193	weigh	t u p u	[t | ts | tʃ] o p o	t o p o (- n - t i)	‘to weigh’
21-201	worm	ʈʂ e n a	ʂ ɨ n a	ʂ ɨ n a	‘worm’
ext_3	arrow	p i s a	p [i | ɨ] s a	p i a ^3^	‘arrow’
ext_14	call	i w a r a	[i | ɨ] ɽ a n a	ɽ a n a (- t i) ^2,5^	‘to call’
ext_29	earth (soil)	u w a	o ɽ a	o ɽ a ^1^	‘cultivated field’
ext_53	heavy	b i kʷ e	β [i | ɨ] [k | w] ɨ	i w ɨ ^8^	‘heavy’
ext_65	louse	b i a	β [i | ɨ] a	i a ^8^	‘louse’
ext_66	maize	ʃ i k e	s [i | ɨ] k i	ʂ ɨ k i ^6^	‘maize’
ext_85	palo santo	a n a n i	[a | hi] n a n [i | ɨ]	n a n ɨ ^4^	‘wito (ceremonial)’ ^D^

The segments are grouped into evolving units^39^, with segments in square brackets separated by pipe indicating an individual segment with one-to-many correspondences in the predictions (see Table 2).

Crucially, some correspondence patterns only arise as phonetically regular when taking the new cognates into account. This expectation was spelled out explicitly in the methodological considerations from the Stage I protocol. The correct predictions from the extended set that are relevant to the new correspondence patterns are presented alongside the main results table. The full list of correct predictions in the extended set, including those which are not directly relevant to the new correspondence patterns, are provided as part of the Supplementary Material.

1. Correspondence of Proto-Takanan */w/ to Proto-Panoan */ɽ/
2. Proto-Takanan */ɽ/ corresponds to Shipibo-Konibo /n/
3. Loss of intervocalic fricative
4. Loss of initial [ a | hi ] (with three-syllabic words)
5. Loss of initial [ i | ɨ ]
6. Correspondence of Proto-Takanan */ʃ/ to Shipibo-Konibo /ʂ/
7. Correspondence of Proto-Takanan */e/ to Proto-Panoan */i/
8. Loss of initial /β/ before /i/, possibly in Proto-Panoan
  A. Colexification bitter and sour: https://clics.clld.org/edges/887-1906
  B. Colexification pain and disease: https://clics.clld.org/edges/1783-1986
  C. Colexification grandfather and grandson: https://clics.clld.org/edges/1383-1618. In related Panoan languages the form still means grandfather.
  D. Both Palo Santo (Bursera graveolens) and Wito (Genipa americana) are trees used for ceremonial purposes.

There is ample evidence that the newly described correspondence patterns reconstruct to Proto-Panoan and indeed describe correctly predicted cognates. Pattern 1 is attested through several established reconstructions for Proto-Panoan, such as */ɽ ɨ/ for nose. Likewise, the patterns 3 and 7 also have several examples that are reconstructed to Proto-Panoan, like */p i a/ ‘arrow’ and */ʔ i a/ ‘louse’. In the latter, the glottal stop in Proto-Panoan seems to be a remnant of Proto-Takanan */β/. The same is true for /i w ɨ/, which reconstructs as */ʔ  i w ɨ/^15^. This provides even more plausability to the loss of the initial consonant in Shipibo-Konibo. The fact that the remaining patterns reconstruct to Proto-Panoan is shown through examples from the Northern branch of Panoan languages. The Northern branch (Matses, Matis, Korubo) is the first branch of the family to split off the remaining languages and is considered to be more conservative in some regards^17^. The presence of a cognate in this branch and another is generally taken as a validation for the reconstructability of a form to Proto-Panoan^15^. For the proposed cognate from pattern 4 and 7 (/m ɨ n i/, 14-127), Matses has /m e n e/^38^. For the proposed cognate from pattern 2 (/n i n i/), Matses has a form /n i n-/ ‘to pull dragging’^17^, with the final vowel being dropped, a common pattern in Matses. Those examples show that the predicted forms attested in Shipibo-Konibo are indeed cognate to the reconstructed forms in Proto-Takanan and reconstruct along the proposed patterns to Proto-Panoan. Pattern 8, the loss of word-initial β, seems to be restricted to predicted occurrences of /β/ before /i/: examples ext_53 and ext_65 show this pattern, while /β/ before /a/ does not drop (9-81, 17-163). For the loss of segments in specific positions observed in other patterns (3, 4, 5), the conditioning context is unclear and needs to be further investigated, but several examples confirm the validity of the pattern nonetheless. We further observe that the correspondence of Proto-Takanan */i/ to Shipibo-Konibo /ɨ/ occurs exclusively after the retroflex phonemes /ɽ/ and /ʂ/. This seems to be a specific phonological context in which this pattern occurs. In other cases, the correspondence of Proto-Takana */i/ in Shipibo-Konibo is /i/.

The newly observed correspondence patterns 1, 2, and 7 are directly related to the reconstructed phoneme inventory of Proto-Pano-Takanan, since they establish new sound correspondence patterns beyond established contexts. First, the retroflex tap that is reconstructed for Proto-Panoan emerges as corresponding to Proto-Takanan */w/, in addition to already have been proposed to correspond to Proto-Takanan */d/. The fact that both */d/ and */w/ are reconstructed for Proto-Takanan and show cognates with Proto-Panoan in identical contexts suggests that both were also part of the phoneme inventory of Proto-Pano-Takanan. Second, Shipibo-Konibo /n/ corresponds to all of */n/, */r/, and */ɽ/ of Proto-Takanan in identical contexts. Taking correspondence patterns of other Panoan languages into account, this suggests that **/r/ and **/ɽ/ merged into */r/ in Proto-Panoan, and subsequently */r/ and */n/ merged into /n/ in Shipibo-Konibo. Third, Proto-Takanan */e/ seems to correspond to /ɨ/ in Shipibo-Konibo in some cases (14-127, 16-132). The fact that the vowels from Shipibo-Konibo reconstruct to Proto-Panoan^15^ suggests that there were such correspondences between both Proto-languages as well, which makes the proposal of an additional vowel in Proto-Pano-Takanan necessary. The remaining sound correspondence patterns that are newly described mostly refer to the loss of a sound in specific contexts (‘merge with /ø/’). Those cases should be analyzed more closely to narrow down the specific phonological contexts in which they occur.

As becomes clear from the newly observed correspondence patterns, three new phonemes have to be proposed for Proto-Pano-Takanan: **/ɽ/, **/w/, and **/ɨ/. All three have at least two cognate sets in our correct predictions that showcase such patterns. A merge of two phonemes (**/r/ - **/ɽ/, and **/w/ - **/d/) is proposed in Proto-Panoan, since no phonological context can be identified that would warrant a split. In the case of **/e/, a merge is proposed in Proto-Takanan of **/e/ and **/ɨ/ to */e/. The relevant correspondence patterns are summarized in Table 7, together with their reflexes in Matses from the Northern branch.

**Table 7:** New correspondence patterns make it necessary to propose additional proto-phonemes for Proto-Pano-Takanan.

Proto-Pano-Takana	Proto-Takana	Proto-Pano	Matses	Shipibo-Konibo
**n	*n	*n	n	n
*r	*r	*r	n	n
**ɽ	*ɽ	*r	**n**	**n**
*d	*d	*ɽ	d	ɽ
**w	*w	*ɽ	**d**	**ɽ**
*ɨ	*e	*ɨ	ɨ	ɨ
**e	*e	*i	**i/e**	**i**
*i	*i	*i	i	i

The bold rows refer to the new observations.

## Discussion

We have found considerable further evidence that strengthens the proposal of a Pano-Takanan language family. For the specific case of our prediction set, we have found 42 exact phonetic matches with a solid semantic link between them. Of these, more than 20 come from the core set, surpassing the established threshold from the pre-registration. We consider this strong evidence in favor of a genealogical relation between Panoan and Takanan languages. The observation of new sound correspondence patterns between Proto-Takanan and Proto-Panoan also leads to the proposal of three new phonemes for Proto-Pano-Takanan. This shows that through the combination of different methods (elicitation and comparison of basic concepts, computational prediction, elicitation of predictions), we cannot not only evaluate the proposal of genealogical relationships between language families, but also find new sound correspondence patterns that contribute to the reconstruction of proto-languages.

The correct predictions that fulfill criteria of regularity are complemented by a set of possible matches that include phonetic irregularities or semantic deviations that need further consideration. These do not count towards our main evaluation threshold, but might be considered for future analysis. Those cases are presented in Table 8. The relevant deviations are again marked by superscript symbols:

**Table 8:** Possible matches between predictions and attested forms without full phonetic regularity or confirmed colexifications.

ID	Concept	Proto-Takana	Prediction	Form	Meaning
2	announce	kʷ ei s a	[k | w] ɨ [i | ɨ] s a	k ɨ ʃ a (- n - t i) ^Z^	‘announce’
16	blow of wind	w u	ɽ o	ʂ o (- n a - t i) ^Z^	‘blow (of person)’
73	thin (slim)	kʷ e s a	[k | w] ɨ s a	k ɨ a ^3^	‘hidden’ ^E^
79	go out	kʷ i n a	[k | w] [i | ɨ] n a	w i n a (- t i)	‘to row’ ^F^
97	join	z i t a	ʃ [i | ɨ] [t | ts| tʃ] a	ʃ i t a (- t i)	‘to cross (a river)’ ^F^
105	long, tall	ʃ u n u	s o n o	ʃ o n o ^Z^	‘lupuna (tree sp.)’ ^G^
157	shout	k e k e	k ɨ k ɨ	k ɨ k ɨ	‘nightmare’ ^E^
171	swell	ʃ e k e	s ɨ k ɨ	s ɨ k ɨ	‘broken’
ext_17	cassava	kʷ a w e	[k | w] a ɽ ɨ	k a ɽ ɨ	‘sweet potato’
ext_280	old	z i r i	ʃ [i | ɨ] n[ i | ɨ]	ʂ ɨ n i ^Z^	‘old, used’

These are not counted to the general evaluation of the hypothesis.

E. Can’t judge colexification, since meaning is not part of CLICS3.
F. Boats are the main form of movement along the rivers where the speakers of Shipibo-Konibo live, the relation between movement verbs to a more specific form related to water is thus not surprising for riverine people like some Panoan speaking groups. The Takanan are non-riverine people.
G. One of the tallest trees in the Amazon, often with huge cultural importance.
Z. Phonetic irregularity in fricative that is not covered by established sound correspondence patterns.

Some of the examples fit well in the observed patterns, but cannot be evaluated due to a lack of entries in CLICS. For example, example 73 presents another case of intervocalic vowel loss. However, hidden is not part of Concepticon, and thus not of CLICS either. Similarly, for example ext_17, the concepts are not part of CLICS. However, we can query the Lexibank dataset^40^ and can find two cases in which a language shows colexification of cassava and sweet potato, namely Sawila (Timor-Alor-Pantar, sawi1256) and Keuw (isolate, kehu1238). Even though we do not count this towards the items relevant for the threshold, it should probably be considered in future reconstructions. Other cases (79, 105, 157) are semantically plausible, but cannot be evaluated at the current state.

An important question for the reconstruction of Proto-Pano-Takanan that could not be solved in this study are the correspondence patterns of fricatives, which is also shown in the above table. In contrast to the seven correspondence patterns for fricatives tentatively proposed for Proto-Pano-Takanan by Girard^16^, in V&Z there is only evidence for two such patterns between Proto-Takanan and Proto-Panoan: */ʈʂ/ to */ʂ/, and */j/ to */s/, respectively. For both cases, we find additional evidence in our prediction set. We also find two cases where Proto-Takanan */ʃ/ corresponds to Shipibo-Konibo /ʂ/. In summary, this means that we have no clear correspondence patterns for Proto-Takanan */z/ and */s/, and neither do we have them for Proto-Panoan */ʃ/. None of the possible correspondence patterns of fricatives that emerge in the table of deviations was found more than once. This is probably the most difficult task in the reconstruction of Proto-Pano-Takanan right now.

One further recurring pattern are the loss of intervocalic fricatives (2-15, ext_3). However, we also observe some cases where the intervocalic fricative does not drop. We argue that those cases might be due to avoidance of homophony^41^. Considering for example the contrast between /p i a/ ‘arrow’ and /p i s a/ ‘toucan sp.’, we observe that the loss of the intervocalic fricative would result in the same form /p i a/. Similarly, the loss of the fricative in /i s a/ ‘porcupine’ would result in the same form as /i a/ ‘louse’. If this analysis is correct, then we can also order the sound changes, since the loss of the word-initial */β/ would have needed to occur before the loss of the intervocalic fricative.

The only newly proposed correspondence pattern that cannot (yet) be reconstructed to Proto-Panoan is the loss of word-initial [ i | ɨ ] (Proto-Takanan */i/). There are several reasons to pay close attention to this pattern and to take it into consideration. The first necessary observation is that there are various cases in which an initial /i/ is not dropped. This could suggest that the merger with */ø/ is restricted to */ɨ/. In the sample of predicted cognates, this correspondence pattern is restricted to two cases, both of which refer to transitive verbs. It is important to highlight that most nouns in Proto-Takanan have an absolutive prefix */e-/, which would also correspond to */ɨ/ in Proto-Panoan. However, this prefix is not attested in Proto-Panoan. A possible explanation for both patterns is that (transitive) verbs in Proto-Pano-Takanan were also marked with an absolutive prefix **/ɨ-/. While in Proto-Panoan this marker would have disappeared completely along with the prefix on the nouns, it would have been fossilized in Proto-Takanan, losing its grammatical function (in case it was still productive in Proto-Pano-Takanan). This seems like a reasonable explanation, but is nothing more but a hypothesis at the moment that needs to be verified or rejected in upcoming reconstruction projects of Proto-Pano-Takanan. Two equally plausible hypothesis of course would be that a) the forms are not actually cognate and the resemblance is pure coincidence, or b) that the loss of initial vowels is triggered by some other mechanism, in parallel to the loss of initial [ a | hi ]. Crucially, more occurrences of the correspondence pattern in question need to be found that reconstruct to Proto-Pano and are cognate with Proto-Takanan.

Methodologically, establishing cognate reflex predictions makes the assumptions behind sound correspondence patterns explicit. This enables us to test not only the correspondence patterns themselves, but also the genealogical relation between languages. Scholars can apply our method in several scenarios. An ideal use case would be a small amount of initial evidence for a genealogical relation between languages based on possible cognates in a list of basic vocabulary. This provides the researcher with a concrete workflow: a) establish possible cognates based on a limited list of basic vocabulary, b) extract the sound correspondence patterns suggested by the possible cognates, and c) test the hypothesized relationship on a larger sample of data. In some cases, this could help to uncover further cognates which are hidden through semantic change. In cases where there is no genealogical relation between the languages, we do not expect a significant number of correct predictions. Clear-cut criteria and the explicit prediction of sound changes that are established in advance help establishing an unbiased judgement of the results.

## Protocol registration

The registered protocol can be found on OSF: https://doi.org/10.17605/OSF.IO/FGBM7. The predictions used for elicitation are presented in another pre-registration on OSF: https://doi.org/10.17605/OSF.IO/VAY2G. The data collection began before in-principle acceptance of the Stage I protocol, but after the submission. The reason were time-constraints related to the fieldwork in Pucallpa, Peru. No changes have been made to the predictions after the submission of the protocol.

## Data Availability

The annotated results table is shared here: https://github.com/pano-takanan-history/cognate-prediction. For the pilot study, we have used the publicly available datasets of Proto-Panoan (https://github.com/pano-takanan-history/oliveiraprotopanoan, v1.2.0)^15,36,42^ and for Proto-Pano-Takanan (https://github.com/pano-takanan-history/valenzuelazariquieypanotakana, v1.0.0)^9,43^. For the creation of the predictions, we made use of our digitization of a dataset for Proto-Takanan (https://github.com/pano-takanan-history/girardprototakanan, v1.0.0)^16,44^.

## Code availability

The code used for the predictions is shared here: https://github.com/pano-takanan-history/cognate-prediction.
